# Food production and agricultural systems on the southwestern frontier of the Han Empire: archaeobotanical remains from the 2016 excavation of Hebosuo, Yunnan

**DOI:** 10.1007/s12520-023-01766-9

**Published:** 2023-05-04

**Authors:** Wei Yang, Zhilong Jiang, Alice Yao, Rita Dal Martello, Jieming Jiang, Huomin Xie, Xuexiang Chen

**Affiliations:** 1 School of History and Archives, Yunnan University, Kunming, 650118 Yunnan China; 2 Yunnan Provincial Institute of Cultural Relics and Archaeology, Kunming, 650021 Yunnan China; 3 Department of Anthropology, University of Chicago, Chicago, IL 60637 USA; 4 Department of Archaeology, Max Planck Institute of Geoanthropology, Kahlaische Str. 10, 07745 Jena, Germany; 5 Jinning Archaeological Workstation of Yunnan Provincial Institute of Cultural Relics and Archaeology, Jinning, 650605 Yunnan China; 6 Joint International Research Laboratory for Environmental and Social Archaeology, Shandong University, Jinan, 250100 Shandong China

**Keywords:** Dian, Han Dynasty, Wheat, Rice, Irrigation, Chenopodium

## Abstract

Dian Basin in Yunnan province is an important center for both early agricultural production and centralized state formation. Settled agricultural villages are present in the province since at least the third millennium BC, and by the first millennium BC, the Dian Culture, a highly specialized bronze polity, flourished in the Dian Basin and surrounding area, until it was conquered by the Han in 109 BC. The increased deployment of flotation at recent archaeological excavations in Yunnan allowed the reconstruction of agricultural practices from the Neolithic to the early Bronze Age, documented at Baiyangcun, Haimenkou, and Xueshan among others. However, archaeobotanical evidence relating to the pivotal period right before and after the Han conquest have so far been lacking, with only limited written records about agricultural production in the Shiji by Sima Qian. Here we present for the first time direct archaeobotanical evidence relating to this transitional period as revealed by rich Han period deposits found during the 2016 excavation of Hebosuo, the largest Dian settlement investigated in Yunnan so far, dated by direct AMS on charred cereal grains and artefactual evidence as spanning from between 850 BC–220 AD. Following the Han conquest, the main components of the agricultural system did not undergo radical changes, but the weedy flora indicates a heavier reliance of wet-land rice systems, evidencing a higher level of water management or even irrigation practices, and the consequent intensification of the agricultural production. These findings on shifting agricultural regimes in Yunnan also contribute to current debates about the interplay between intensification, food risk, and ecology in times of political instability.

**Supplementary Information:**

The online version contains supplementary material available at 10.1007/s12520-023-01766-9.

## Introduction

The study of archaeobotanical remains from imperial frontiers is critical to understanding how premodern empires managed agricultural intensification under varying socio-environmental conditions as well as the impacts of empire building associated with those endeavors (Marston 2015, Rosenzweig and Marston 2018, Hastorf 2017, Morrison 2018). During the last two centuries BC, the Han state in China undertook a series of imperial campaigns that extended its territorial reach across parts of Eurasia and Southeast Asia, leading to a continental empire comparable in size to Rome at its apex. While the footprint of Han expansionism has been documented by recent excavations of farming colonies and irrigation systems along the proto-Silk roads in northwest China, the significance of the southern frontier — a region characterized by rugged terrain and pronounced monsoonal variability — to Han economic history remains poorly understood despite historiographic accounts of large-scale agrarian projects. Located in Southwest China, the Dian Kingdom arose during the later 1st millennium BC and was on the periphery of the region from which the Han Dynasty emerged. The Dian are historically significant for being among the earliest complex polities in Southwest China, competing with the early Chinese Empire before being conquered by the Han in 109 BC (Zhang 1997; Allard 1998; Qian 1956). After this, Dian dominions were reorganized into 24 counties that together constituted the Yizhou prefecture, a newly established political unit that was placed under the administration of a centrally appointed governor (Ban 2005; Li and Chen 2021). In addition to establishing political control, the Han also implemented a massive migration of people from the central plains to the Dian Basin (Lin 2005). The end of the first millennium BC was thus a pivotal time in Yunnan for cultural and social change. Most scholars have either approached the development of sociopolitical hierarchies through the study of bronze production, especially as evidenced in élite burials (Dewall 1967; Lee 1996; Tawara kanji 2001; Rode 2004; Chiou-Peng 2004; Yao 2005) or questions regarding the local impact of Han imperialism and migration-driven population growth from a funerary perspective (i.e., Yang 2011; Chiang 2012; Wu 2018; Yao 2016). In recent years, less mundane aspects of Dian society have also started to gain more research attention, including the investigation of the food production system through the systematic collection and analysis of environmental remains, including flotation for the retrieval of ancient plant remains (Yao et al. 2015; Yang et al. 2021; Dal Martello et al. 2021). However, little direct evidence for discussing the history of agricultural production is available for the Bronze Age to the Han imperial period, or the transition between the fall of the Dian and the establishment of the Han rule over Central Yunnan.

In this study, we draw on archaeobotanical evidence from directly dated assemblages obtained through flotation during the 2016 excavation of the site of Hebosuo, a key settlement associated with the Dian and representing occupations before and after Han conquest. Hebosuo is one of the few early, large-scale settlement sites dating to the first millennium BC that have been systematically investigated so far in the Dian Basin and surrounding areas, Central Yunnan. By documenting food production at an important southern frontier with well-dated archaeobotanical deposits from Hebosuo (YPICRA in prep), we address how agricultural intensification was pursued, that is, either through the adaptation or change to local Bronze Age crop regime, and whether new systems of land use were involved. Our findings provide a reconstruction of the historical ecology of an imperial frontier, showing how the tracing of continuities and changes in cereal cultivation and field management strategies, especially in relation to irrigation practices, may enrich comparative discussions of anthropogenic impacts of empire building. These findings on shifting agricultural regimes in Yunnan also contribute to current debates about the interplay between intensification, food risk, and ecology in times of political instability.

## The site of Hebosuo

### Environment, excavation history, and chronology of Hebosuo

**Fig. 1:**
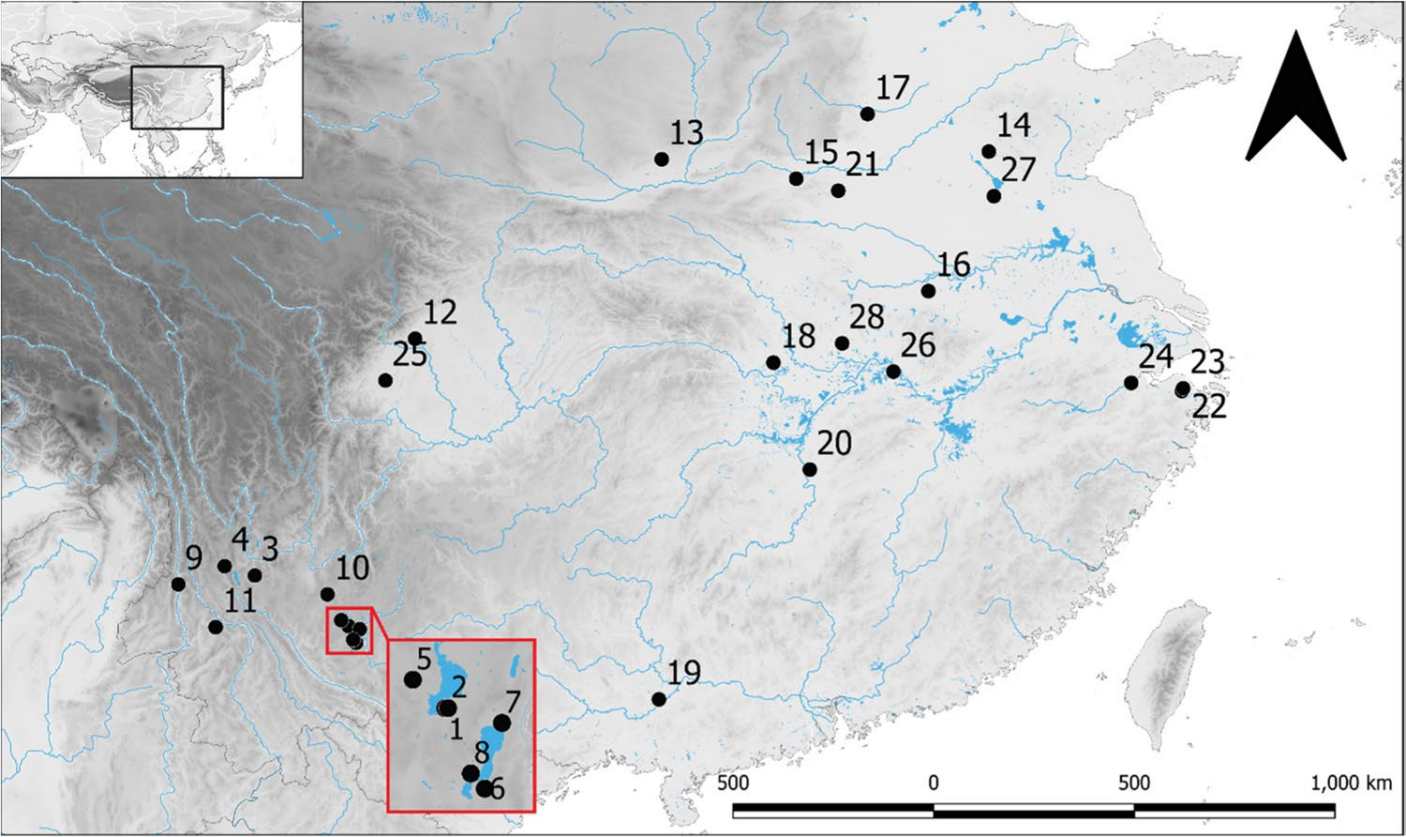
Map showing location of Hebosuo and other sites mentioned in text: 1, Hebosuo; 2, Shizhaishan; 3, Baiyangcun; 4, Haimenkou; 5, Dayingzhuang; 6, Guangfentou; 7, Xueshan; 8, Lijiashan; 9, Shilinggang; 10, Yubeidi; 11, Yingpanshan; 12, Guiyuanqiao; 13, Xiahe; 14, Zhuguo; 15, Erlitou; 16, Gushi; 17, Yinxu; 18, Baoshan; 19, Luopowar; 20, Mawangdui; 21, Peiligang; 22, Hemudu; 23, Tianluoshan; 24, Kuahuqiao; 25, Pujiang; 26, Echeng; 27, Guishan; 28, Shuihudi. Made with QGIS 3.16.1

**Fig. 2:**
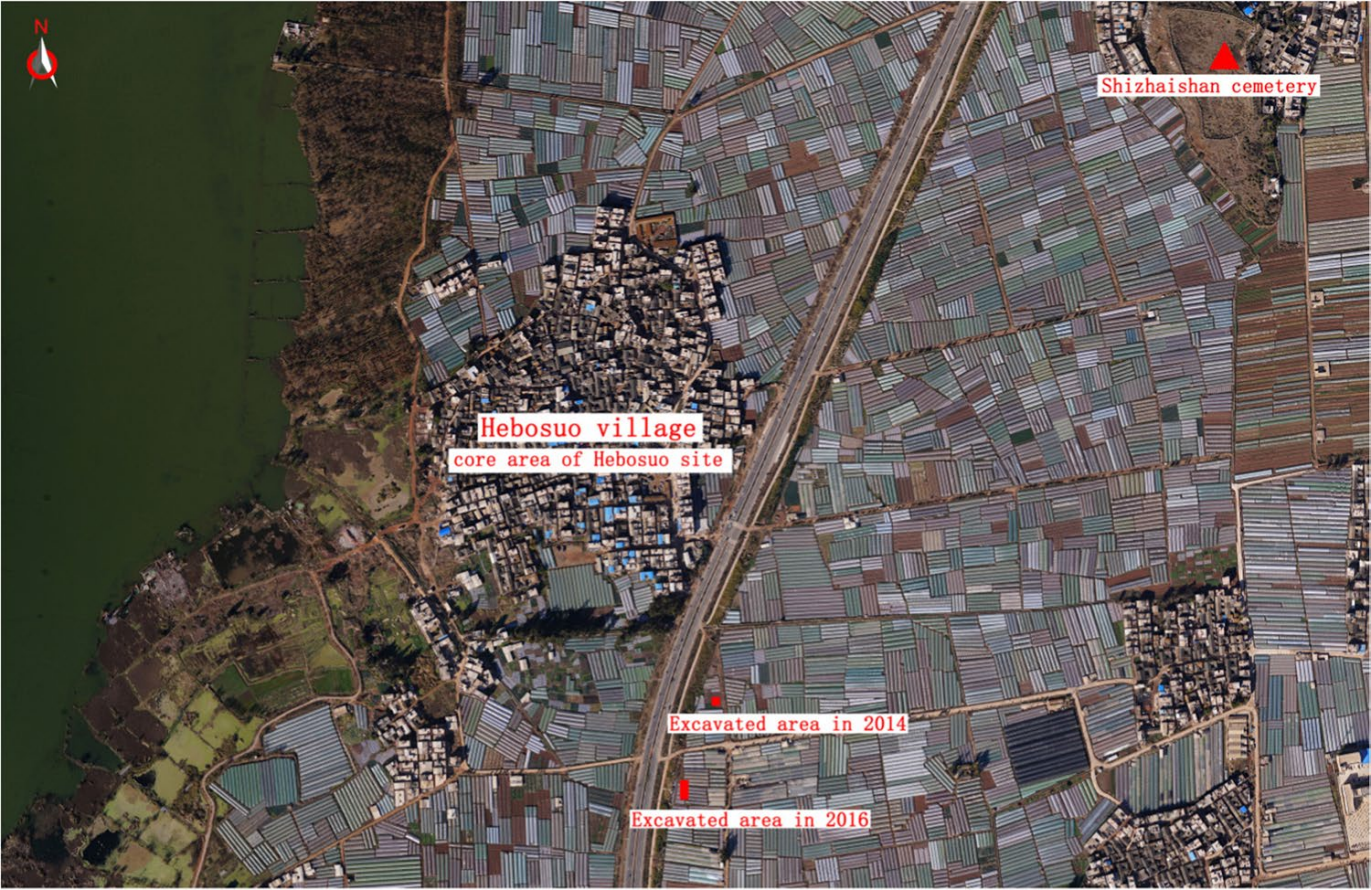
Birds’eye view of Hebosuo surroundings, with indication of modern village, excavation areas for 2014 and 2016 campaigns, and Shizhaishan cemetery. Photo by YPICRA

Hebosuo is located nearby the Shangsuan town in the Jinning district on the eastern bank of Lake Dian, about 60 km from Kunming (102.244212 E; 24.25246 N; Figs. 1 and 2), and only 1 km south from Shizhaishan cemetery, the earliest discovered site belonging to the Dian Culture, or Dian Kingdom, formerly known as Shizhaishan Culture (Wang 1980; Jiang 1998). It is the largest of a group of 16 sites known as the “Hebosuo cluster,” dated to the first millennium BC, which were identified over the course of two joint Sino-American archaeological surveys led by the Yunnan Provincial Institute of Cultural Relics and Archaeology (henceforth YPICRA) in association with the Department of Anthropology University of Toronto in 2008 and 2012. On these occasions, the eastern, southern, and western banks of the lake were surveyed (YPICRA 2012; YPICRA et al. 2014) with the goal of investigating the settlement patterns of Dian sites, and Hebosuo was identified as one of the core settlements of the Dian (Yao et al. 2015), which continued to be occupied after the Han arrival in 109 BC.

Lake Dian is the largest water reservoir of Yunnan province. It has an area of over 330m^2^ and a total length, north to south, of 40 km. The Dian Basin has elevations ranging between 1885 and 1900 m above sea level and is characterized by a subtropical monsoonal climate, presenting general mild, warm, and humid conditions. The favorable weather makes Kunming known as the city of the eternal spring, and the Dian Basin presents no harsh cold winter or hot summers. Average annual temperature is attested between 14.7 and 15.4℃, with sunlight hours total to 2302–2345 per year. Average annual precipitation is attested on 900 mm. These environmental conditions and relatively flat terrain are extremely favorable to agricultural production, allowing up to four cropping per year with irrigation; today cultivated crops include rice, wheat, rapeseed, and broad bean (Kunming Jinning District Local Chronicles Comittee 2003). The Dian Basin in central Yunnan is considered among the most important centers for agricultural production in the province since prehistoric times and paleo-environmental reconstruction established that current weather conditions stabilized in the region at the beginning of the 1st millennium BC (Xiao et al. 2020; Dearing et al. 2008; Shen et al. 2005; Dykoski et al. 2005; Hillman et al. 2019).

Following the 2008 and 2012 surveys, the YPICRA and the Department of Anthropology of the University of Chicago led the first excavation season of Hebosuo in 2014. The principal aim of the excavation campaign was to establish a precise chronology for the site through direct radiocarbon dating (YPICRA et al. 2019; Yao et al. 2020). In 2016, a second excavation campaign was undertaken with the aim of defining precise cultural phases and especially targeting post-Dian period cultural development at the site.

**Fig. 3:**
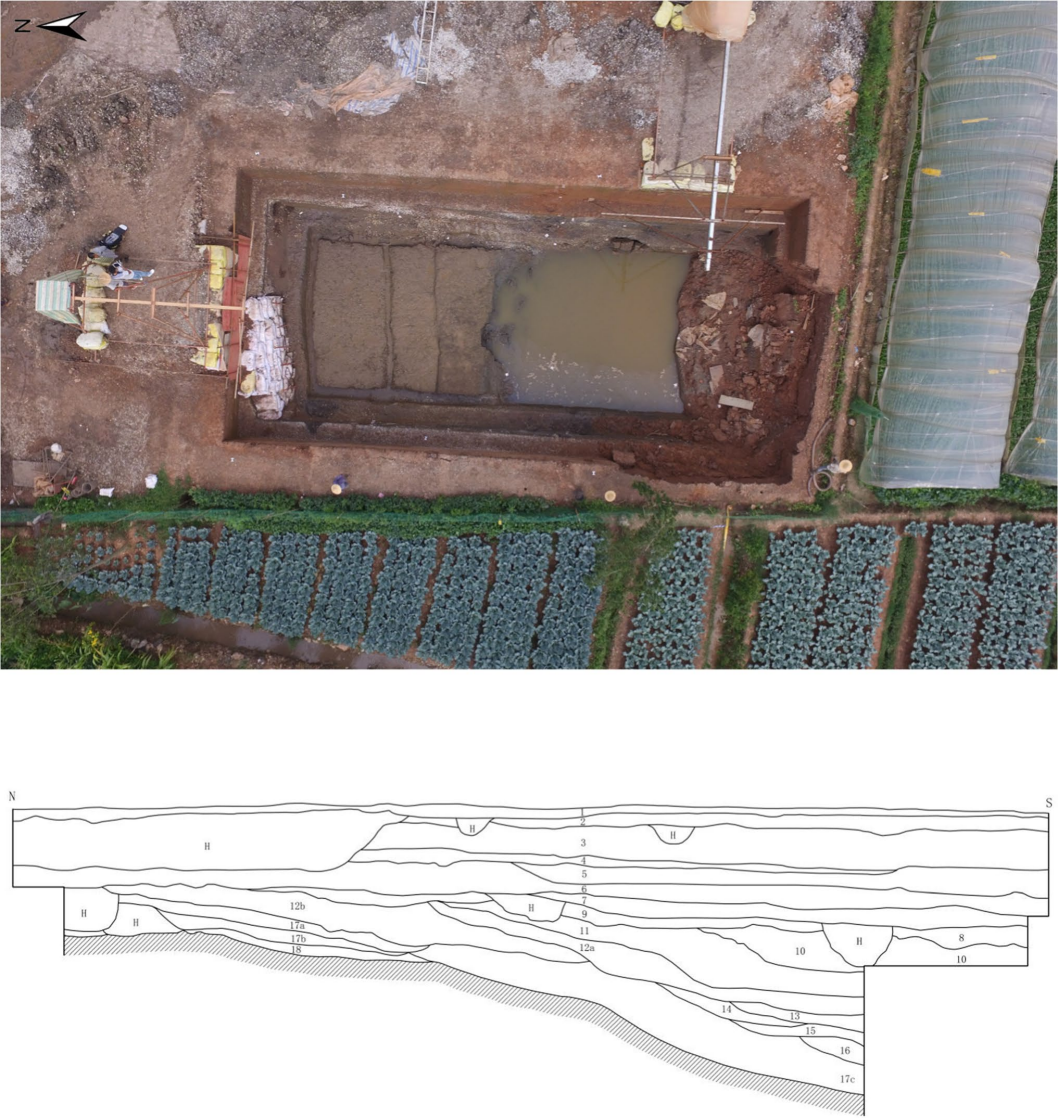
Top: Aerial photo of the 2016 excavation trench of Hebosuo, showing groundwater level emerging during excavation, photo by YPICRA. Bottom: Profile section of 2016 Hebosuo excavation trench, east side

Both excavation campaigns focused on the southeast area of the site, the 2016 trench was located about 200 m south from the 2014 trench (Fig. 2). The 2016 excavation area comprised of two 10 × 10 m trenches. A total of 19 cultural layers were individuated during excavation, reaching a depth of about 5 m (Fig. 3). Only two features were revealed during the 2016 campaign, H1 and H2, representing two ash pits; in addition to those pits, 5 scattered postholes were also individuated, possibly indicating an architectural feature. Both pits and postholes were located underneath the 11th stratigraphic layer. Additionally, further rubbish pits were individuated at the end of the excavation on the western wall profile of the trench; these were not identified in the course of the excavation due to the due to the high groundwater levels (Fig. 3). While the 2014 excavation revealed mostly Dian period deposits, and a continuous occupation from c. 1200 BC to c. 500 BC, corresponding to the late Bronze Age period pre-Dian to the Dian period in Yunnan (YPICRA 2019); the 2016 excavation revealed rich late Dian and Han period deposits and related material culture, c. 800 BC–220 AD.

**Fig. 4:**
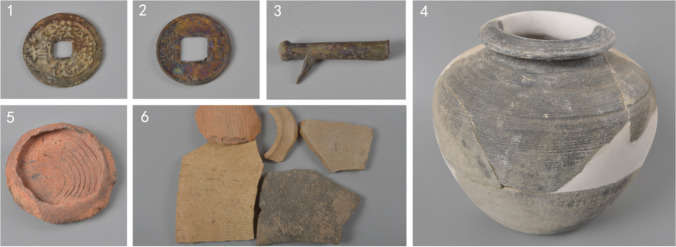
Artefacts recovered from the 2016 excavation of Hebosuo. 1, *Honghua Tongbao* coin from layer 5; 2, *Wuzhu* coin from layer 15; 3,“*gaigongmao*,” component of chariot from layer 16; 4, Han style *guan* jar from layer 11; 5, Dian ceramic saucer from layers 17 to 19; 6, Han tiles from layers 7 to 16

**Fig. 5:**
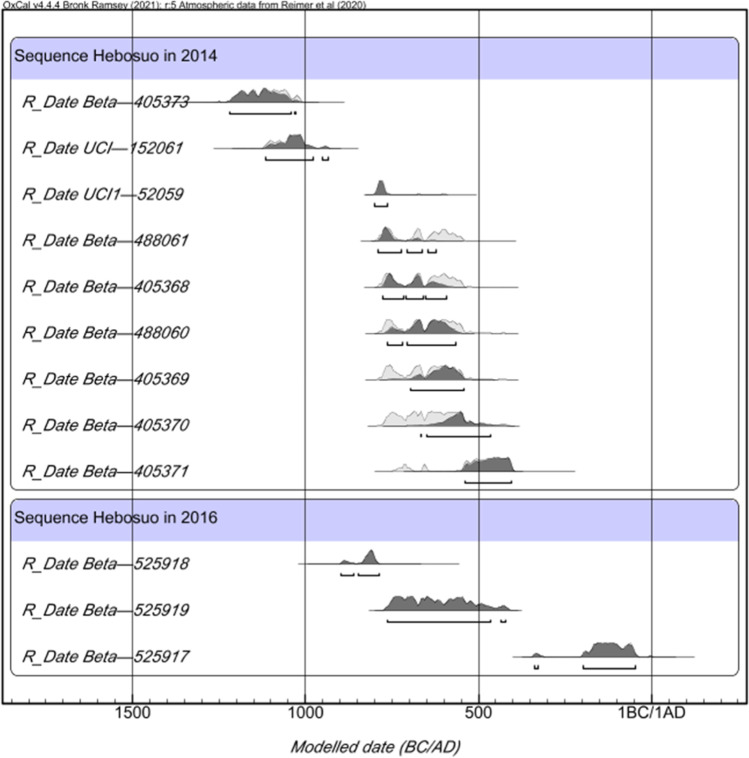
Bayesian model of radiocarbon dates from Hebosuo, including 2014 and 2016 excavation seasons. Made with OxCal 4.4.4 (Bronk Ramsey 2009)

On the basis of stratigraphic sequence and artefactual evidence (Fig. 4) as well as direct radiocarbon dating on charred cereal grains (Table 1 and Fig. 5), the following chronology was established for the 2016 Hebosuo excavation:

- layers 1 to 6: Ming-Qing Dynasties (1368–1911 AD)
- layers 7 to 16: Han Dynasty (202 BC–220 AD)
- Layers 17 to 19: Dian Kingdom (Dian or Shizhaishan Culture) (800–109 BC)

**Table 1:** Radiocarbon dates from Hebosuo, with indication of context of provenance, material dated, and lab code. 2014 dates from Yao et al. (2020) and Yang et al. (2021)

Context	Material	Lab code	Cal. date BP	Calibrated date (Bayesian model agreement index)
2016 excavation				
2016JHT1⑯-1	Two wheat grains	Beta-525917	2110 ± 30 BP	204–46 cal BC (95.40%)
2016JHT1⑰c-1	Three rice grains	Beta-525918	2660 ± 30 BP	859–794 cal BC (85.7%)
2016JHT1⑱-1	Four rice grains	Beta-525919	2470 ± 30 BP	768–476 cal BC (95.40%)
2014 excavation (from Yao et al. 2020;Yang et al. 2021**)**				
3a	Wheat grain	Beta-404368	2520 ± 30	651–544 cal BC
3b	Wheat grain	UCI1-52059	2565 ± 20	806–748 cal BC
4a	Rice grain	Beta-405369	2510 ± 30	786–541 cal BC
4b	Wheat grain	Beta-405370	2490 ± 30	775–514 cal BC
5	Wheat grain	Beta-405371	2410 ± 30	550–399 cal BC
5	Wheat grain	Beta-405360	2510 ± 30	786–541 cal BC
6	Rice grain	Beta-405361	2530 ± 30	651–544 cal BC
6	Rice grain	UCI-152061	2870 ± 20	1126–929 cal BC
7	Rice grain	Beta-405273	2920 ± 30	1214–1016 cal BC

Layers 17 to 19 contained numerous Dian culture artefacts and were largely consistent in time with layers 3–6 yielding Dian cultural deposits revealed in the 2014 excavations to the northwest (Table 1) (Yao et al. 2020). These temporal correspondences indicate contemporaneous occupations in both areas of Hebosuo during the Bronze Age. The precise phases between layers 16 and 1 of the 2016 excavation were strongly based on the relationship between the stratigraphic sequence and artefactual evidence. This includes the large quantity of Han artefacts recovered between layers 16 and 7, such as corded decorated tiles, *gaigongmao*, a typical Han style chariot implement, coins bearing *wuzhu*, which were produced from 118 BC, under Emperor Wudi, and in use until the end of the Eastern Han Dynasty (220 AD). From layer 6 onward, instead, numerous typical Ming and Qing Dynasty styles tiles have been recovered, as well as a copper coin bearing *Honghua Tongbao* (corresponding to 1679-1681 AD) recovered in layer 5, thus indicating that layers 6 to 1 were occupied in more recent times, during the Ming and Qing Dynasties.

### Previous archaeobotanical work at Hebosuo: pre-Dian and early Dian agriculture

Hebosuo is a shell-midden site, its location close to the Dian Lake indicates that in the past the lake fluctuating water levels might have affected the deposition and preservation of the plant remains. Flotation analyses at Hebosuo were first undertaken during the 2014 excavation season of Hebosuo, which focused on the occupation of the site between c. 1200 BC and c. 500 BC. The archaeobotanical analyses revealed a rich assemblage showing an agricultural system based on the mixed cultivation of dryland (wheat and millets) and wetland crops (rice), as well as contributions from soybean; large quantities of *Chenopodium* and few *Prunus* type fruits possibly collected locally (Yang et al. 2021). Cereals crops constitute the main component of the 2014 archaeobotanical assemblage of Hebosuo, and among the cereals, wheat is prevalent, followed equally by rice and millets (Yang et al. 2021).

## Material and methods

A total of 57 archaeobotanical samples were collected for flotation during the 2016 excavation of Hebosuo. The majority of the archaeobotanical samples came from stratigraphic cultural layers; the strata were distinguished by differentiating colors and soil inclusions. Samples for flotation were taken from securely identified layers, as well as from the two rubbish pits. The samples analyzed covered a stratigraphic sequence from layer 19 to layer 7, corresponding to the late Dian and Han Dynasty periods. One sample was also collected from the upper layers 6–1 representing Ming-Qing Dynasty periods deposits; since this sample was sterile and these later time periods were not the main focus of the campaign, no further samples associated with these strata were collected. The total volume of soil floated was 599 L, and each sample had an average bulk soil volume of 10.5 l. Four additional samples (T1⑬:1HP, T1⑭:1HP, T1⑭:2HP, T1⑯:1HP) were taken where large quantities of botanical remains became visible with the naked eye during excavation; these samples were not floated since they consisted of only large botanical remains and have been reported separately below (Tables 2, 3, 4, and 5, see also Supplementary Material Table S1). The samples were processed at the site through manual bucket flotation, the float was collected using a 0.18-mm mesh size sieve, and the organic material was poured onto cotton bag and let to dry naturally in the shade. All samples were sorted with a Leica M125c low-power binocular microscope at magnification up to × 100, and the sorting was undertaken by WY at Joint International Research Laboratory for Environmental and Social Archaeology, Shandong University, as part of her doctorate research. Macro-botanical remains were extracted, counted, and identified following the Flora of China (eFloras 2008) nomenclature. Photos of the remains were taken with the Zeiss Stemi 200C camera and processed (including measurements of the main crop species) with ProgRes Capture Pro V2.8 software.

**Table 2:** Summary of Hebosuo 2016 archaeobotanical assemblage; total plant remains include unidentified remains

Chronology	No. of samples	Recovery rate	Tot liters floated	Preservation status	Tot recovered plant remains	Density item (L)
Han	45 floated	100%	495	Charred	64734	130.7
202 BC–220 AD				Waterlogged	590	1.19
	4 handpicked	na	na	Charred	40	na
				Waterlogged	263	
Dian	11 floated	81.8%	97	Charred	3243	33.3
800–109 BC				Waterlogged	8	0.08

**Table 3:** Summary of charred macro-botanical remains from Hebosuo

	Dian	Han
	Layers 17–18 (800–109 BC)	Layers 7–16 (202 BC–220 AD)
Volume floated (L)	97	495
No. of samples	11	45
Cultural layers	11	40
Ash pits	-	4
Cereal crops		
* Triticum aestivum* — “I” elongated wheat grains	8	22
* Triticum aestivum* — “I” immature grains	3	1
* Triticum aestivum* — “II” compact wheat grains	15	99
* Triticum aestivum* — II wheat grains	-	1
* Triticum aestivum* — rachises	11	7
* Oryza sativa* — rice grains	18	41
* Oryza sativa* — rice immature grains	1	2
* Oryza sativa* — rice grain fragments (> 1/2)	9	25
* Oryza sativa* — rice immature grain fragments (> 1/2)	1	13
* Oryza sativa* — rice grain fragments (< 1/2)	2	8
* Oryza sativa* — rice immature grain fragments (< 1/2)	-	6
* Oryza sativa* — spikelet bases	2830	61302
* Setaria italica* — foxtail millet grains	4	13
* Setaria italica* — foxtail millet immature grains	2	-
Cerealia	5	9
Cultivated legumes		
* Glycine max* — soybean	-	3
* Vigna angularis* — adzuki bean	-	2
Possible cultivated species		
* Fagopyrum esculentum* — *buckwheat*	1	2
* Chenopodium album*	63	409
* Chenopodium ficifolium*	181	2258
Fruits, nuts, and tubers		
* Vitis* L. grape	-	1
* Duchesnea indica* — Indian strawberry	-	10
* Crataegus cuneata* — hawthorn	-	4
Indet. fruits or nuts	2	6
Indet. tubers	-	2
Other possible economic species		
* Zanthoxylum bungeanum* Sichuan pepper	3	7
Seeds of field weeds		
Seeds of dryland cultivation		
* Polygonum lapathifolium*	7	37
* Polygonum lapathifolium* var. *salicifolium*	21	16
* Digitaria* sp.	-	2
* Setaria viridis*	-	4
* Elsholtzia ciliata*	2	-
* Kochia scoparia*	-	9
* Xanthium strumarium*	-	-
* Stellaria media*	3	4
* Rorippa globosa*	1	-
* Panicoideae* A. Br	-	2
Seeds of wetland cultivation		
* Polygonum amphibium*	-	6
* Polygonum hydropiper*	-	1
* Schoenoplectus juncoides*	4	69
* Scirpus triqueter*	1	92
* Pycreus flavidus*	-	3
* Cyperus iria*	1	-
* Eleocharis* R. Br	1	41
* Bulbostylis* Kunth	9	6
Weeds from uncultivated land		
* Polygonum capitatum*	1	2
* Rumex hastatus*	-	1
* Rumex nepalensis*	6	-
* Vicia sepium*	1	1
* Thermopsis lanceolata*	-	7
* Paspalum scrobiculatum*	2	1
* Lophatherum gracile*	1	-
* Clinopodium chinense*	-	1
* Perilla frutescens*	-	1
* Stachys sieboldii*	1	2
* Atriplex patens*	-	65
* Sigesbeckia orientalis Linnaeus*	-	5
* Oxalis corniculata*	6	29
* Verbena officinalis*	1	18
* Malvastrum coromandelianum*	-	1
* Nicandra physalodes*	-	1
Weeds of uncertain habitat		
Nymphaeaceae	-	1
Cyperaceae	-	1
Violaceae	2	1
Unidentified	13	48
Unidentifiables	-	3
Total macro-remains (excluding unidet)	**3230**	**64683**

**Table 4:** Summary of waterlogged macro-botanical remains from Hebosuo

	Dian	Han
	Layers 17–18 (800–109 BC)	Layers 7–16 (202 BC–220 AD)
Volume floated (L)	97	495
No. of samples	11	45
Cultural layers	11	40
Ash pits	-	4
Fruits		
* Lagenaria* Ser. bottle gourd	-	6
* Prunus persica* peach	-	119
* Cerasus* Mill. cherry	1	-
* Prunus mume* Japanese plum	-	3
* Prunus* L	-	3
* Rubus* L	-	380
Seeds of field weeds		
Seeds of dryland cultivation		
* Actinostemma tenerum*	1	6
Seeds of wetland cultivation		
* Oenanthe benghalensis*	-	1
* Potamogeton* L	3	10
Seeds of wasteland		
* Zehneria indica*	2	1
* Sambucus javanica*	1	10
* Potentilla bifurca*	-	55
Seeds of arboreal plants		
* Celtis* L	-	2
Total macro-remains	**8**	**590**

**Table 5:** Summary of archaeobotanical remains from handpicked samples T1⑬:1HP, T1⑭:1HP, T1⑭:2HP, T1⑯:1HP

Samples	T1-⑬:1HP	T1⑭:1HP	T1⑭:2HP	T1⑯:1HP
Charred remains				
Cultivated crops				
* Triticum aestivum* wheat mature grains type I	-	-	1	-
Fruits				
*Vitis* L	-	-	1	-
*Crataegus cuneata* Sieb. et Zucc	1	-	-	-
Tubers	-	-	-	1
Seeds of field weeds				
Seeds of dryland field weeds				
* Polygonum lapathifolium* var. *salicifolium* Sibth	-	-	2	-
Seeds of wasteland weeds				
* Thermopsis lanceolata* R. Br	1	-	-	-
Unidentified remains				
Indet type 3	2	-	-	30
Indet type 4	1		-	-
Waterlogged remains				
Fruits				
* Lagenaria* Ser	42	-	30	3
* Prunus persica* L	-	160	-	-
* Prunus mum*e Sieb	-	5	-	1
* Prunus* L	1	2	-	-
* Cerasus* Mill	-	1	-	-
Seeds of field weeds				
Seeds of dryland cultivation				
* Xanthium strumarium* L	-	-	4	2
Seeds of wetland field weeds				
* Oenanthe benghalensis* Benth. et Hook. f	1	-	1	-
Seeds of wasteland weeds				
* Zehneria indica (*Lour.)Keraudren	4	-	2	-
Other weedy remains				
Asteraceae	-	-	1	-
Arboreal crops				
* Choerospondias axillaris* (Roxb.) B. L. Burtt & A. W. Hill	-	1	-	-
* Pinus* L	-	-	-	2

## Results

### General features of the assemblage and key economic taxa

The archaeobotanical samples from Hebosuo presented a very rich assemblage comprising of both charred and, in minor quantity, waterlogged remains, including mostly wood fragments, and seeds and peach pits (see Tables 2, 3, and 4). A total of 67,977 individual charred remains and 598 waterlogged remains from 34 families and 60 species were recovered from the 56 floated archaeobotanical samples (Tables 3 and 4). This includes whole seeds, seeds fragments, rice spikelet bases, and wheat rachises, as well fruit stone and tuber fragments. Unidentified and unidentifiable categories include those remains that could not be identified, or had no characterizing feature that allowed identification (Supplementary Material S1). From the four handpicked samples, a total of 303 individual plant remains were recovered (Table 5). These have not been included in the quantitative analyses below, but are presented in supplementary material Table S1; a summary is also provided in Tables 2 and 5 below.

The samples from the 2016 excavation season of Hebosuo had a high density of charcoal fragments; for the ≥ 1 mm fraction, charcoal content per each sample averaged 6.4 g/L, with the highest recorded density of 36.6 g/L of floated soil (from sample t1 h1-1). A higher presence of charcoal fragments correlated with a higher presence of ancient charred plant remains. The average density of charred seeds per floated soil liter was 130.7 for Han period samples and 33.3 for Dian period samples (Table 2). Waterlogged remains showed a lower density per liter of floated soil (Table 2). There is a strict correlation between the quantity of charcoal and charred seed present in each sample; the higher the quantity of charcoal fragments found corresponds to a higher number of charred seeds found. This indicates that the majority of the seeds became incorporated in the archaeobotanical assemblage following the burning of domestic waste, such as left-over food after cooking or the disposal of crop processing waste products. Waterlogged remains, instead, are mostly represented by peach pits; this might indicate that the fruits were consumed in a different way than other retrieved food remains, and became incorporated into the archaeobotanical assemblage through post-depositional underground water movements. Ancient hydrological variation studies in the area attest to several events of water table levels rise across the millennia (Yan et al. 2020), and this could explain the presence of both charred and waterlogged remains at the site, given the location of Hebosuo near to the lake.

**Fig. 6:**
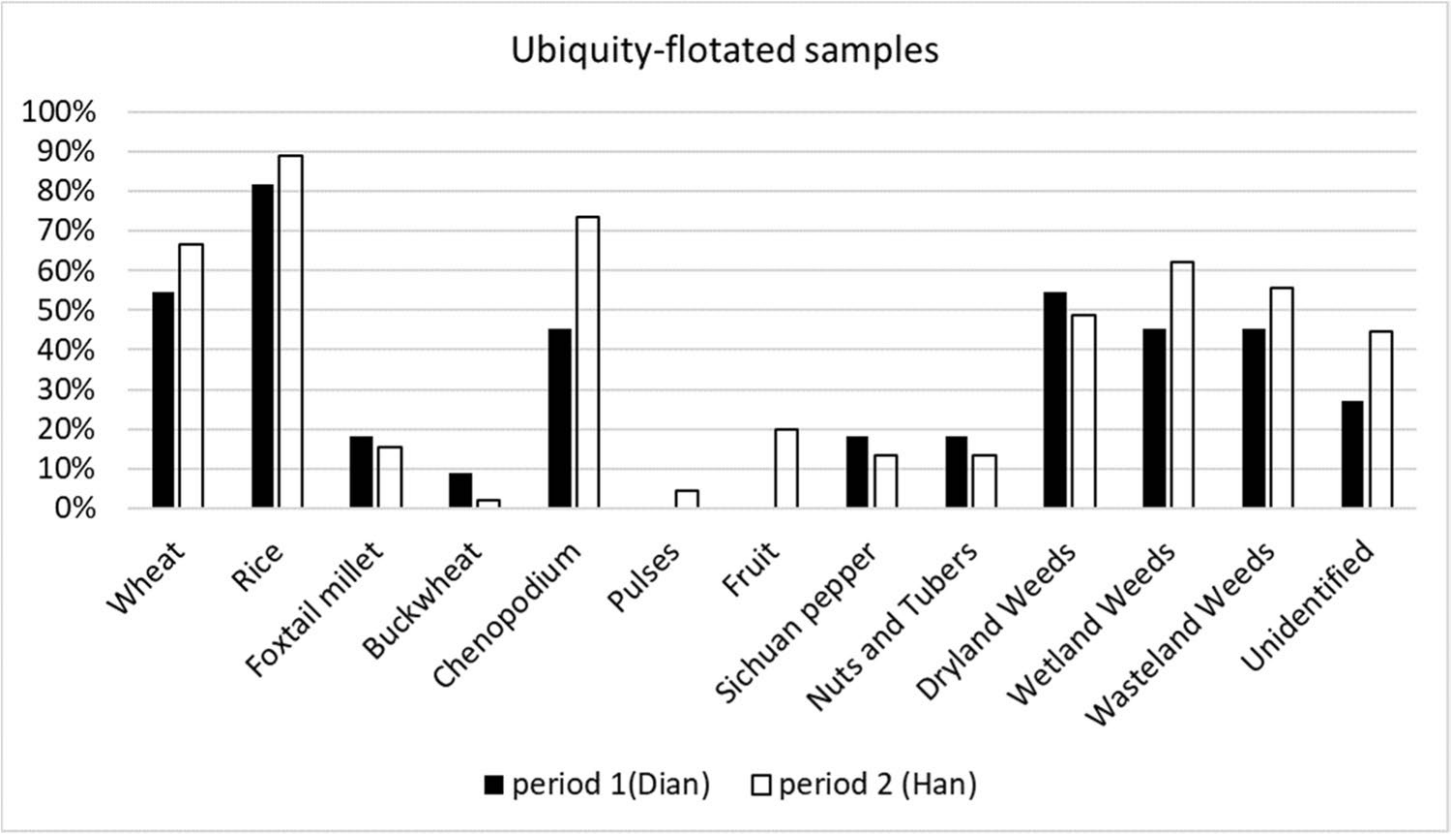
Ubiquity of archaeobotanical charred remains grouped in main categories from the 2016 Hebosuo samples. Total number of samples analyzed per period: Dian = 11; Han = 45

**Fig. 7:**
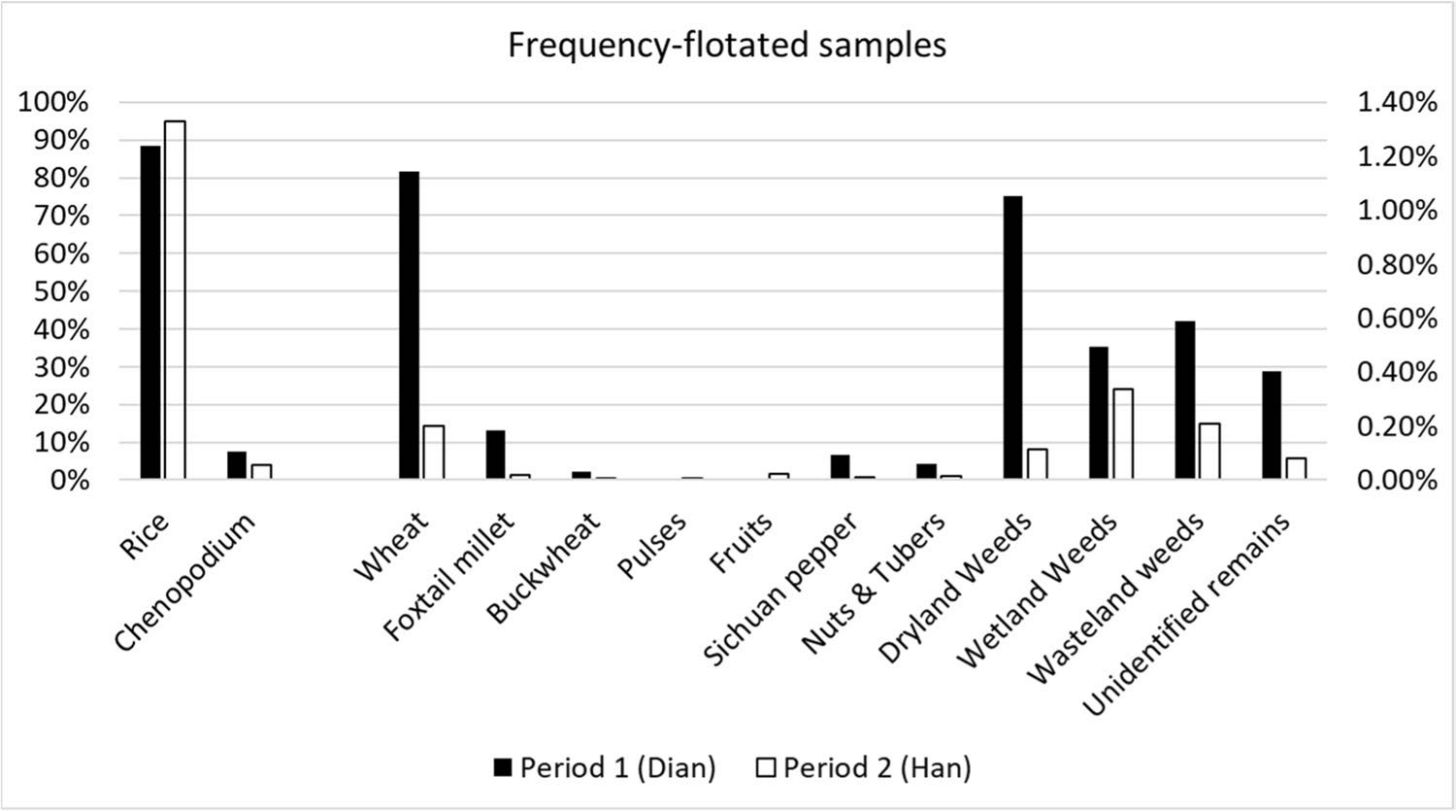
Frequency of archaeobotanical charred remains, including grains and chaff, grouped in main categories from the 2016 Hebosuo samples. Rice and Chenopodium are plotted on the left on a 100% scale, the remnants of the archaeobotanical remains are plotted on the right with a separate scale. Total number of samples analyzed per period: Dian = 11; Han = 45; total number of identified remains per period: Dian = 3236; Han = 64722

Ancient plant remains were divided in the following categories: cereal crops, cultivated legumes, other possible economic species, fruits nuts and tubers, and weedy taxa. Cereals and weedy taxa remains show the highest ubiquity across both periods of occupation, as well as the highest absolute counts, after *Chenopodium* seeds (see below, Figs. 6 and 7). Pulses, fruits, and other possible economic species are present in lower quantities among the charred remains; however, they represent the majority of the waterlogged material, indicating they still had an important role in in the overall economy of the site (Figs. 6 and 7 and Tables 2 and 3).

### Cereal crops

Three species of cereals were found in the 2016 Hebosuo samples, and these include in order of importance free-threshing wheat (*Triticum aestivum*), rice (*Oryza sativa*), and foxtail millet (*Setaria italica*). Both grains, whole and fragmented, as well as rice spikelet bases and wheat rachises (Tables 3 and 4; supplementary material Table S1; Fig. 9). Some of the cereal remains were too fragmented and badly preserved to allow for precise identification, and these have been grouped into the Cerealia category. For the quantitative analyses below, only grains were counted, excluding Cerealia, rachises, and rice spikelet bases. Additionally, grain fragments were equaled to approximate whole grain counts, for rice fragments bigger than half grains, these were counted as 1; for fragments smaller than half grains, these were counted as 3 fragments = 1 grain. In Tables 3 and 4, numbers are estimated of whole grains equivalent.

#### Rice — *Oryza sativa*

Rice remains recovered from the 2016 Hebosuo samples included whole grains, both mature and immature, fragments of grains, and spikelet bases. The rice fragments were further categorized by size, those fragments that were bigger than half grain were counted as 1 grain, fragments smaller than half grain were instead approximated to whole grain count estimates, with 3 fragments counting as 1 grain. In total, 65080 rice remains where retrieved, including 126 grains and 64954 spikelet bases; all of the spikelet bases were of the domesticated type. Chronologically, rice remains increase both in ubiquity, from 81.8 to 88.9% from the Dian to the Han period, and in frequency with rice remains representing 88.4% of the total recovered archaeobotanical remains in the Dian samples and increasing to 94.9% in the Han samples (Figs. 6 and 7). Rice grains appeared generally well preserved by charring; each grain showed a crescent-shaped embryo on the side, with clear longitudinal ridges along the grain. Especially the longitudinal ridges are clearly visible even in fragments of rice grains, and this allows for their inclusion in the rice category. Those grains which were shrunken, with no clear longitudinal ridges along the grain, presenting a concave embryo have been categorized as immature grains through the comparison of ancient and modern immature rice grains.

**Fig. 8:**
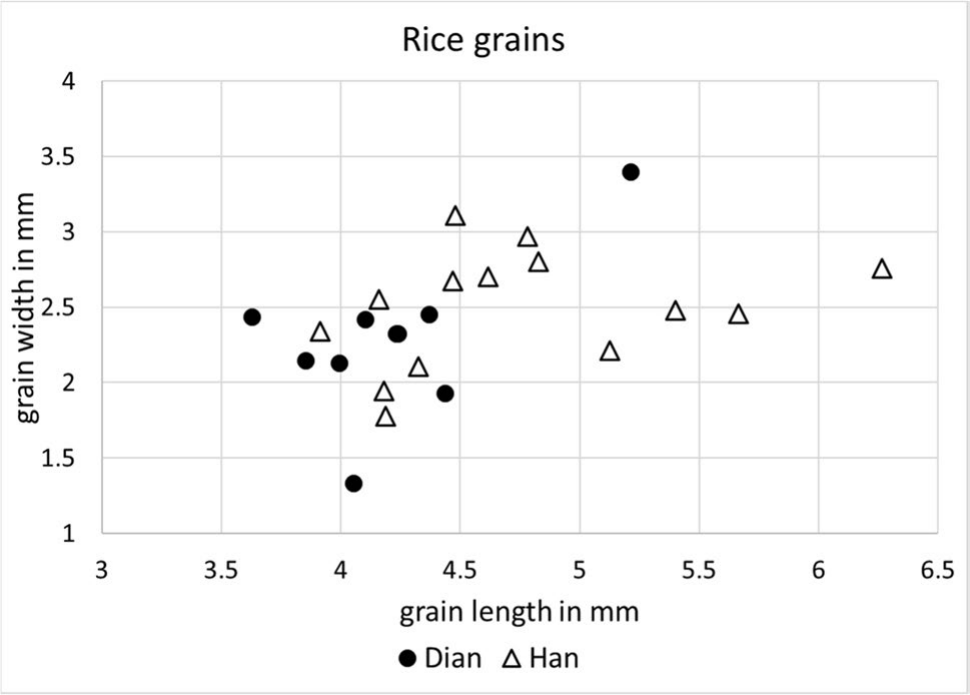
Scatterplot of rice length and width measurements from 2016 Hebosuo

Twenty-five whole, well-preserved mature grains were selected for measurements (Fig. 8; Supplementary Material S2), average length was 4.52 mm with a standard deviation of 0.6 mm, average width was 2.40 mm with a standard deviation of 0.4 mm, and average thickness was 1.93 mm with a standard deviation of 0.3 mm. L:W ratio was on average 1.9 (Fig. 8). According to previous morphometric research on rice grains, those grains having a L:W > 2.2 belong to the so-called *indica* type (Harvey and Fuller 2005; Castillo et al. 2016) and grains with a L:W < 2 belong to the *japonica* type. Most of the measured rice grains from the 2016 Hebosuo samples showed a L:W < 2; only 5 of the measured grains had a L:W > 2.2, and 3 belonged to the middle range 2–2.2; this indicated that rice from Hebosuo can be identified as *Oryza sativa* subsp. *japonica.* This is in line with previous rice remains found from earlier sites in Yunnan, such as Baiyangcun (Dal Martello et al. 2018), Haimenkou (Xue et al. 2022), and Xueshan (Wang et al. 2019).

#### Free-threshing wheat — *Triticum aestivum*

Wheat remains include whole grains (*n* = 150) and rachis fragments. These were found in samples dating to the both the Dian and the Han periods, showing an increased ubiquity from Dian to Han from 54.5 to 66.7%; however, this corresponds to a slight decrease of overall frequency (Fig. 7). In general, these seeds had no visible husk, the embryo was sunken, and the ventral furrow was clearly visible; the back of seed showed the typical rounded lower hump. Additionally, a few grains were significantly smaller, stunted, with a shrunken embryo and overall shrunken surface of the grain. These were identified as immature grains. Due to highly pronounced morphological differences between some of the wheat grains, these were further categories in the following sub-types:

- type I: larger size grains with an elongated shape, a total of 35 grains belonged to this type;
- type II: highly compact grains with an overall rounder profile; smaller in overall size than type I; a total of 115 grains belonged to this type.

**Fig. 9:**
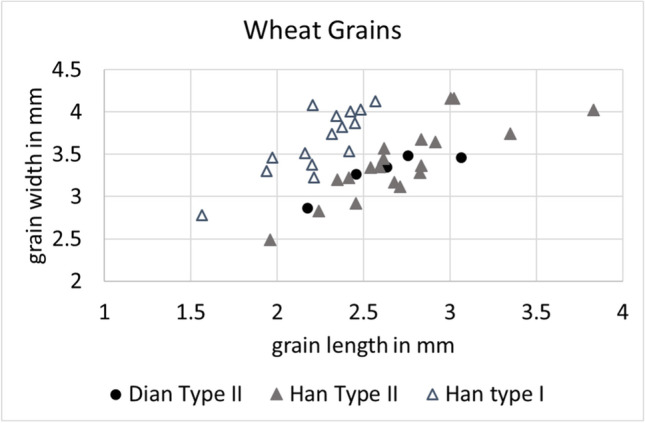
Scatterplot of length and width measurements of wheat grains from 2016 Hebosuo

Both types were recovered from both time periods. Thirty-nine well-preserved and whole, mature grains where selected for measuring, and morphometric measurements further supported the division of wheat grains in the two subtypes: 15 type I grains were measured and their average L:W ratio was 1.63; 24 type II highly compact wheat grains showed instead a much smaller average L:W ratio of 1.25 (see supplementary material table S2). Overall, type II grains were on average 3.38-mm long and 2.7-mm wide (see Fig. 9). Due to the lower quantity of wheat grains retrieved from the Dian samples, only 5 grains belonging to this time period could be measured.

#### Foxtail millet — *Setaria italica*

Foxtail millet was not present in large quantities among the samples analyzed. A total of 19 grains were recovered from 9 samples, representing the 15.8% of the samples analyzed. Chronologically, millet remains show a decrease trend in both ubiquity and frequency, from 18.2 to 15.6% in ubiquity, and c. 0.2% to slightly less than 0.02% in frequency from the Dian to the Han period (Figs. 6 and 7). Morphologically, millet grains are round or slightly oval in shape, the embryo has a clear reversed U-shaped depressed area, and the embryo is as big or longer than half of the total length of the grain. Eight well-preserved mature millet grains were selected for measuring and averaged 1.17 mm in length, 1.05 mm in thickness, and 0.83 mm in thickness (Supplementary Material S2); this is comparable to other domesticated foxtail millet remains from earlier archaeological sites in Yunnan, such as at Baiyangcun (Dal Martello et al. 2018) and Haimenkou (Xue et al. 2022). There was no clear size difference between grains recovered from period one to period two of occupation. A few immature grains were also recovered from period 1, and these measured significantly smaller than mature grains; immature grains were on average 0.96-mm long, 0.83-mm wide, and 0.67-mm thick (Supplementary Material S2).

### Cultivated legumes

#### Soybean and adzuki bean

**Fig. 10:**
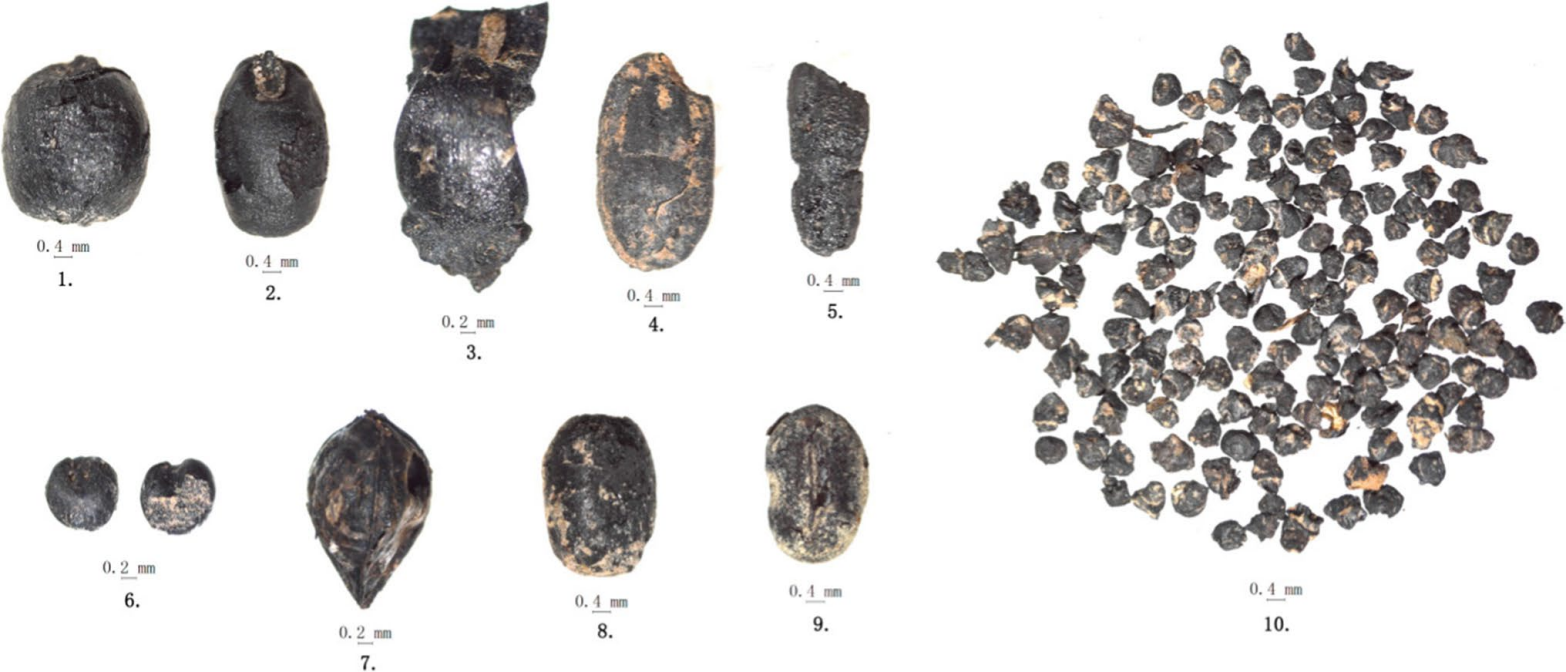
Photos of charred crops from Hebosuo*.* 1, wheat — *Triticum aestivum* type II (highly compact type); 2, wheat — *Triticum aestivum* type I (elongated type); 3, wheat/barley rachises; 4, rice — *Oryza sativa* (mature grain type); 5, rice — *Oryza sativa* (immature grain type); 6, foxtail millet — *Setaria italica*; 7, buckwheat — Fagopyrum cf esculentum; 8, Adzuki bean — *Vigna* cf *angularis*; 9, soybean — *Glycine max*; 10, rice spikelet bases

A few grains of soybean (*Glycine max*) and adzuki bean (*Vigna* cf *angularis*) were recovered from samples belonging to the Han period. Three complete soybean grains were recovered from 2 samples and 2 grains of adzuki beans from 1 sample. The soybean presented an overall round-oval shape with an oval hilum on one side; the thin seed coat was still visible on small sections of the grains (Fig. 10). The thickness of the seed coat in soybean is usually taken as a diagnostic trait when distinguishing wild vs. domesticated specimens (e.g., Murphy et al. 2019). One grain was well preserved enough to be measured, and it was 3.56-mm long, 2.35-mm wide, and 1.93-mm thick. The size of this grain is comparable to modern domesticated soybean.

Adzuki beans had a round shape, slightly flat on both sides, showing a long hilum placed on one side. The seed coat of the recovered adzuki bean was smooth and thin. One grain was measured and it was 3.62-mm long, 2.54-mm wide, and 2.37-mm thick. Adzuki beans are not native to China; current evidence indicates this species might have been domesticated in Japan or Korean and brought to mainland China via early connections and exchange via sea possibly at the end of the first millennium BC (e.g., Chen et al. 2019; Lee 2013).

### Possible cultivated species

#### Buckwheat — *Fagopyrum esculentum*

Three grains of buckwheat were recovered from two samples, 1 grain from sample T1⑰a-1 from period 1 and 2 from sample T1⑬-5 from period 2. All three grains were charred, they present a triangular shape with pronounced longitudinal ridges on each side, and embryo is located in the center of the grain (Fig. 10). The examination of the grain in cross-section revealed slightly curving at the edges. Each grain still presented the coat. One grain could be measured and it was 2.72 mm in length, 1.74 mm in width, and 1.65 mm in thickness. The buckwheat from Hebosuo is comparatively smaller than known modern cultivated buckwheat, but its size is comparable to other known archaeological buckwheat seeds, ranging between 2.21 and 3.99 mm in length and 2.43–3.28 mm in width (Yang et al. 2021).

#### Chenopodium

Abundant quantities of *Chenopodium* grains were found in the samples analyzed: a total of 472 *Chenopodium album* and 2439 *Chenopodium ficifolium* grains. These were found in 38 samples, corresponding 66.7% ubiquity. Both species present very similar seeds, differentiating by seed coat patterns; *C. ficifolium* shows hexagonal depressions on the seed coat and a small size, averaging in diameter less than 1.00 mm; *C. album* seeds have obtuse rim margins, shallower seed coat patterns, and larger seeds, usually with a diameter of 1.2–1.5 mm. Both species have black colored seeds. Additionally, both species are edible, including both the grains and the leaves as greens (e.g., Fogg 1983; Tanaka and Nakao 1976). Although uncharred modern Chenopodium grains show a similarly colored seed coat, however, charred grains have a much more brittle seed coat layer, which is easily broken when examining the seeds under microscope. Modern seed coat is tough and not easily broken with simple pressure by the tweezers, and although charred seeds have a similar seed coat colors to modern seeds, their difference in seed coat elasticity makes them easily distinguishable. Chenopodium seeds decrease from the first to the second period of occupation from 7.5 to 4.1% of the total recovered archaeobotanical remains.

### Fruits, nuts, and tubers

**Fig. 11:**
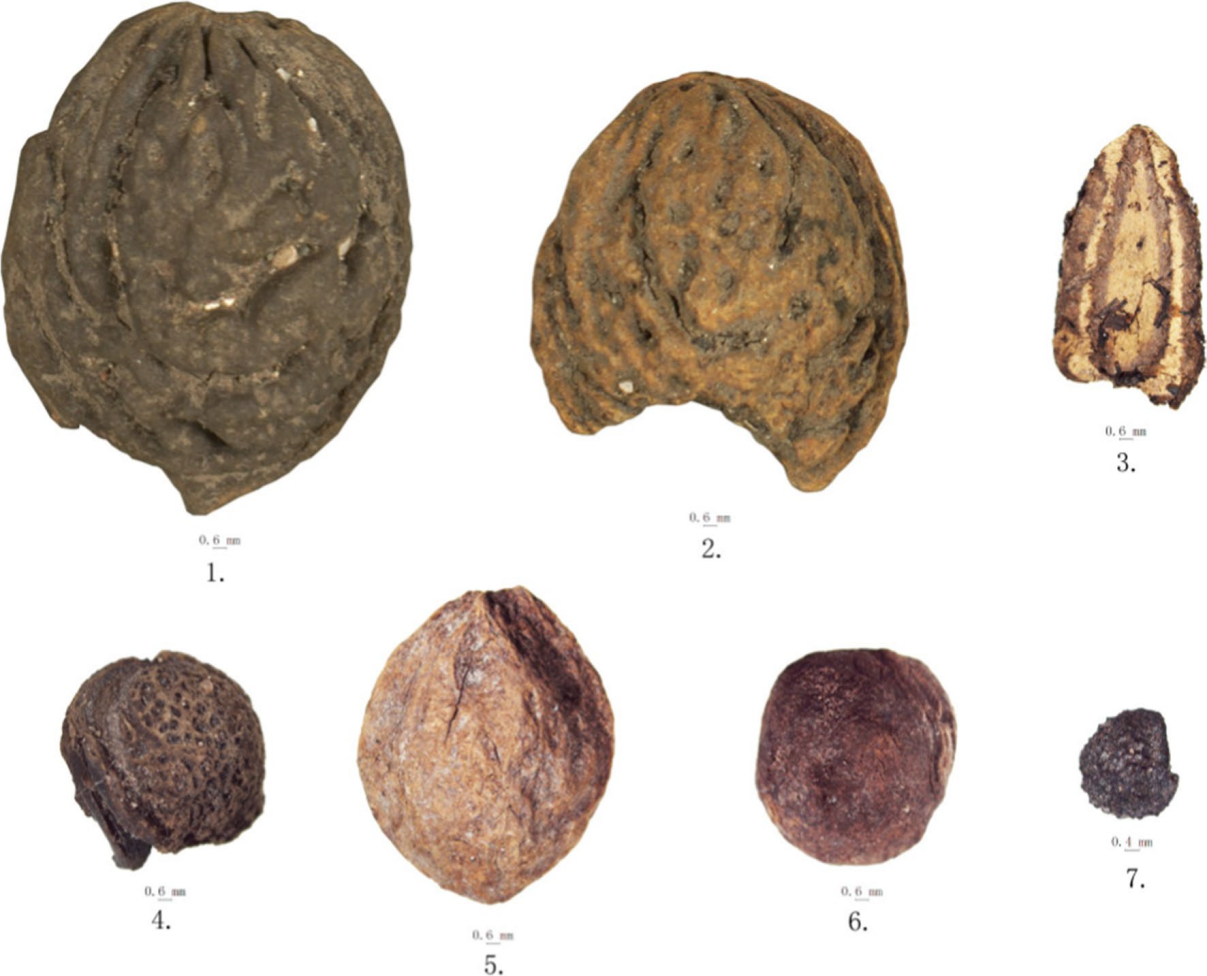
Photos of waterlogged fruit remains and charred Sichuan pepper from Hebosuo. 1–2, peach — *Prunus persica*; 3, bottle gourd — *Lagenaria* sp.; 4, Japanese plum — *Prunus mume*; 5, *Prunus* sp.; 6, cherry — *Cerasus* sp.; 7, Sichuan pepper — *Zanthoxylum bungeanum*

Fruit remains retrieved from the 2016 Hebosuo samples include charred grape seeds (*Vitis vinifera*), hawthorn (*Crataegus cuneata*), Indian strawberry seeds (*Duchesnea indica*), and a few fragments of unidentified fruits and/or nut species, and tubers (Tables 3 and 4; Supplementary Material S1; Fig. 11). Although fruits are present in very low quantities among the charred remains; waterlogged material from the sites is mostly comprised of fruit remains. These include seeds of bottle gourds (*Lagenaria* sp.), several *Prunus* remains, including stones of peach (*Prunus persica*), possible cherry (*Prunus cerasus*), Japanese plum (*Prunus salicina*), and indeterminate *Prunus* stones, as well as *Rubus* seeds (Table 4; Supplementary Material S1). Among the handpicked samples, noteworthy is the find of possible hog plum remains (*Choerospondias axillaris*; Table 5).

Fruit remains account for c. 0.2% of the total recovered archaeobotanical remains, of which 0.19% are Rosaceae type fruits. Although present in both periods of occupation, fruit remains increased considerably in diversity and quantity of remains recovered in the second period of occupation. For example, with the exception of cherry, all other P*runus* and Rosaceae fruits were found in the Han period samples (Tables 3 and 4). Among these, peach stones are the most abundant; due to their large size, these peach pits were mostly collected by hand during excavation from layers 13 to 16. A total of 279 among complete stones and fragments were recovered. Intact stones were measured and are about 1.8–2.2 cm in length and 1.34–1.62 cm in width (See supplementary Material S2 for a complete list of peach stone measurements). Additionally, 186 stones have drill marks at one end, on the surface show concave gaps, and the kernels in the seeds are absent. The extraction of the kernel could be related to detoxification processes to make the pit edible (Hosoya et al 2010).

Three fragments of tuber were found; these are larger in size than cereal grains and have a typical tuber internal structure. All of the tuber fragments were found in samples belonging to the second phase of occupation, but were retrieved only in 5.8% of the samples analyzed.

Some additional unidentified fruit stones were also recovered, but they lacked distinguishable characteristics that could identify them to species. These were found in 10.5% of the samples analyzed.

#### Bottle gourd — *Lagenaria* sp.

A total of 81 bottle gourd (*Lagenaria* sp.) seeds have been recovered from the 2016 Hebosuo samples. All of these were preserved by waterlogging, their appeared creamy yellow in color and presented a narrow and oblong shape, with a wider bottom end, truncated margins, and a protrusion on each side. The top end has a triangular tip, with two longitudinal edges on each side. The hilum is located on the narrow cusp end (Fig. 11). Due to the large dimension of these seeds, the majority of these were collected by hand during the excavation, since they were clearly visible with the naked eye. A small number of them were also recovered through flotation.

### Other possible economic species

#### Sichuan pepper — *Zanthoxylum bungeanum*

A total of 10 seeds of *Zanthoxylum bungeanum* were found in the 2016 Hebosuo samples (three from the Dian samples and four from the Han samples), all of which were preserved by charring. These are of oval shape and slightly flattened at one side, and the surface shows a protruding dotted pattern (Fig. 11). We chose 4 well preserved and complete seeds to measure, and they measured on average 3.3 mm in length and 2.7 mm in width.

### Weedy taxa

A total of 607 seeds of wild weedy taxa have been recovered from the 2016 Hebosuo samples, accounting for 0.89% of the total recovered archaeobotanical remains (Fig. 7). Seeds of field weeds belong overall to 35 different species, which were categorized in dryland, wetland, and wasteland weeds, with the addition of a few indeterminate weeds which could only be identified to family level. This division was made consulting local floras to establish each species growing ecology (eFloras 2008).

Dryland weeds include species associated with dryland cultivation, often found infesting fields of wheat and millet. These include *Polygonum lapathifolium*, *Digitaria* sp., *Kochia scopara*, *Setaria viridis*,* Xanthium strumarium*, and *Stellaria media*. Seeds of dryland weeds decrease in both diversity and abundance from the first to the second period of occupation (Fig. 7).

Wetland weeds comprise those species typically growing in irrigated or more heavily watered agricultural fields, such as paddy rice fields. Wetland weedy taxa recovered from the 2016 Hebosuo samples include *Scirpus triqueter*,* Schoenoplectus juncoides*,* Eleocharis* sp., *Potamogeton* sp., and *Bulbostylis* sp. Wetland weed seeds show a significant increase in presence and variety of taxa from the first to the second period of occupation of Hebosuo.

So-called wasteland weeds are those species associated with disturbed landscapes, growing by hillsides or on abandoned pastoral fields. This includes, for example, *Atriplex patens*,* Potentilla biforcuta*,* Oxalis corniculate*,* Verbena officinalis*, and *Sambucus javanica*. These show an increase in ubiquity through time; however, the overall quantities of wasteland weed type seeds decrease from the Dian to the Han period.

### Arboreal species

Remains of the arboreal species of *Pinus* (pine cone fragments) and *Celtis* (hackberry, two seeds) were also found in the samples analyzed. These were preserved by waterlogging.

## Discussion

### Staple crop composition as evidenced from the archaeobotanical assemblage

The archaeobotanical assemblage retrieved from the 2016 excavation at Hebosuo shows that people inhabiting the site during the mid-first millennium BC practiced a mixed economy based on the cultivation of both dryland and wetland cereal crops, legumes, possibly buckwheat, and *Chenopodium*, and the collection of several local wild fruits, in continuity with earlier reported results from the 2014 archaeobotanical assemblage from the site. Rice was the primary wetland crop, supplemented by wheat and millet, although millet has been retrieved in much lower quantities in comparison to wheat and might have been a secondary crop. The high quantity of rice spikelet bases recovered indicates that rice was grown locally, and the analysis of the associated weedy flora, abundant in wetland species such as *Schoenoplectus juncoide*,* Scirpus triqueter*, and *Potamogeton L.*, suggests that rice was most likely grown in the lowlands, possibly through some sort of water management, and dryland crops were instead grown in the nearby hills.

The high quantity of *Chenopodium* grains retrieved at Hebosuo might indicate that it might have been actively exploited and utilized as food or fodder resource by people living at Hebosuo. *Chenopodium* grains are often reported from early archaeological sites that have undergone flotation analysis, especially from Southwestern China. High quantities of this species have been reported from Yingpanshan (c. 3300–2600 BC, Zhao and Chen 2011) and Guiyuanqiao (c. 3100–1000 BC, SPICRA 2015), in Sichuan, and at the site of Haimenkou (1600–400 BC, Xue et al. 2022) and Guangfentou (c. 800–400 BC, Li and Liu 2016) in Yunnan. At these sites, *Chenopodium* grains were found in high quantities, sometimes representing more than half of the total recovered archaeobotanical remains. At other sites in Yunnan, such as Xueshan, Dayingzhuang, and from the 2014 Hebosuo samples, *Chenopodium* grains are also reported, albeit in much lower quantities (Wang et al. 2019; Dal Martello et al. 2021; Yang et al. 2021). The widespread report of high numbers of archaeobotanical Chenopodium grains from early sites in Southwestern China might indicate that the species was exploited by humans and had some role in the overall production system of the early southwestern China economy. Stems from *Chenopodium* plants have also been known to be used as fire started after appropriate drying. However, how this species was used is difficult to ascertain and future research should address this issue.

Seeds of Sichuan pepper are another common find at sites in Southwest China that have undergone flotation. Today, Sichuan pepper has many uses, it is an important spice in Chinese cuisine, it can be used as anti-septic medicine, for producing beverages, and the woody part of the plant can be used for manufacturing purposes. Sichuan pepper is considered to originate in Sichuan, a neighboring province to Yunnan. In China, the use of *Zanthoxylum* can be traced back to at least the fourth millennium BC, with the earliest reported finds of *Zanthoxylum* seeds from Xiahe, in Shanxi province (c. 3900–3600 BC, Liu et al. 2013). With the increased deployment of flotation in archaeological excavations in the past years, finds of this species have been increasingly reported, with finds from Erlitou (CASS 2014); the ancient city of Zhuguo (Ma et al. 2019); and from several ancient cemeteries including at the Shang Dynasty Gushi cemetery in Henan (Ding 1991), Yachang cemetery at Yinxu (Yang 2012), Chu cemetery in Baoshan, Jingmen, Hubei (BTJRA 1988), Han Dynasty Luopowan cemetery in Guixian county in Guangxi (Cultural Relics Task Force of Guangxi Zhuang Autonomous Region 1982), and at the Han Dynasty Mawangdui tomb in Changsha (HAI and CASIB 1978). Many early sites in Yunnan have also reported finds of this species, including Shilinggang (Li et al. 2016), Dayingzhuang (Dal Martello et al.^2021^), Yubeidi (Yang et al. 2020), and Xueshan (Wang et al. 2019). This shows that *Zanthoxylum* was possibly being exploited during the first millennium BC in Yunnan, especially in the Nujiang, Jinsha, and Dian basins, but the domestication trajectory of this species is still not well understood.

An important find at Hebosuo is the numerous *Lagenaria* seeds. This is a gourd genus native to East Asia, with archaeobotanical finds reported since prehistoric times. In China, bottle gourd remains have been reported from the sites of Peiligang, Hemudu, and Tianluoshan, dating to about 8000–7000 years ago. This indicates that this species could have been domesticated in the lower Yangzi Basin; however, a possible center in Japan has also been indicated as a possibility (Fuller et al. 2010), while a separate American domestication has also been demonstrated (Kistler et al. 2014). In the Americas, ancient gourd finds date to at least 10,000 years ago, and their domestication is usually measured through the progressive thinning of the gourd skin. The fact that gourds remains found at these Neolithic sites presented a morphology closely resembling that of modern day, domesticated gourd, seem to imply that selection pressure was already applied prior to the Holocene (Fuller et al. 2010). Gourds can be used in a variety of ways, many species are edible, and given their thick and sturdy skins, many are used as container once emptied and dried. Gourds depictions have been found in Neolithic pottery remains from China, including the famous Yangshao gourd-shaped bottle (Wang 2014). Additionally, gourds have a highly symbolic meaning in traditional Chinese culture; their shape is thought to resemble female genitalia and therefore bottle gourd is used as fertility (Liu 1996a). In antiquity, gourds were used as offering in Shinto rituals, symbolizing immortality (Liu 1996b). In Yunnan, bronze bottle gourds used as musical instruments, known as *sheng* pipe, and other miniature figurines of bottle gourds have been found in Dian graves at the cemetery sites of Shizhaishan, Lijiashan, and others (YPM 1959; YPM 1975). This indicates the ritual use of the cucurbit beyond its edible function and its importance in Dian music and ritual dances. There exist two types of gourd *sheng* pipe: the straight or the curved pipe; each is played in a different way and represents two different musical instruments (Ge 1987). The modern shape of the bottle gourd might have been selected by the specific needs to achieve for this musical pipe.

That fruits remains from Hebosuo are mostly preserved by waterlogging, in contrast to charred cereals and other economic species, might indicate that fruit remains were not discarded into the fire, but possibly eaten while wandering through the site and their waste discarded spontaneously. *Prunus* and more broadly fruit belonging to the Rosaceae family have been sporadically reported from other early sites in Yunnan, including at Haimenkou (Xue et al. 2022) and Baiyangcun (Dal Martello et al. 2018). However, at all previous sites, fruit remains are present in very low quantities (Dal Martello 2022). The large quantity of especially peach stones from Han period Hebosuo reported here might indicate a more intensive exploitation of this species starting at the end of the first millennium BC with the arrival of the Han to the province who brought with them a higher cultural and subsistence value for these fruits. According to present evidence, the oldest archaeological peach stones have been reported from Kuahuqiao, in the middle and lower Yangzi river basin, dating to at least 7500 year ago (Zheng et al 2014); and peach remains are widely reported from archaeological sites in China by 4000 year ago (Fuller and Stevens 2019) and in India by 3700 years ago (Fuller and Madella 2001). The oldest written reference to peach is found in Shang Dynasty oracle bones inscriptions (Yu 1996); further references are found in the *Shi Jing*, or Book of Songs, the *Huainanzi*, and other early texts dating to the late second millennium BC onward (Jiang 1998). During the Han Dynasty, peach symbolized immortality and possessed the power of warding off ghosts and evil spirits. Peach stones are a common funerary find in Han period graves including at Pujiang in Zhaohua, Sichuan, in Echeng, Hubei, as well as Guishan in Tongshan in Jiangsu, and at Shuihudi in Hubei (Chen 1994). Clay figurines depicting peach tree branches have also been found at the Han Dynasty Mawangdui tomb in Changsha (HPM and HPICRA 2004). However, in the Yunnan-Guizhou Plateau, few peach remains have previously been found. Previous reports include at the Neolithic site of Duliao (c. 4000 BC; Zheng et al. 2014) in Guizhou and at Baiyangcun (c. 2600–1800 BC; YPM 1981) and Haimenkou (c. 1600–400 BC; Xue et al. 2022) in Yunnan. However, only a few stones and stone remains have been reported from these sites, and the peach finds from Hebosuo are the largest archaeobotanical assemblage retrieved from the province so far. The metrics taken on the stones support their identification as cultivated or actively exploited variety, and the holes drilled at the end of the stones could indicate the removal of the kernels for detoxification purposes, but it could also indicate that this fruit had also a decorating or ritual use in addition to its edible use.

Finally, the finds of arboreal species such as *Pinus* and *Celtis* indicate not only that these types of trees were possibly presented in the immediate vicinity of the village, but could also indicate that people living at Hebosuo used their wood as fuel or for manufacturing purposes.

### Continuity and intensification of cereal crop production during the Han

The agricultural system as attested by the retrieved archaeobotanical remains from the 2016 excavation of Hebosuo does not show substantial changes from the Dian to the Han period. Drawing on a historical ecology perspective, we show how imperial rulers incorporated a pre-existing Bronze Age multi-crop base of wet and dry (wheat-millet-rice) cereals that is well-adapted to the monsoonal conditions of the Dian basin. However, even though the same rotation of crops from the Bronze Age was maintained throughout the two periods, our analysis of weed assemblages indicates Han officials attempted to intensify agricultural production by shifting to an irrigated land use system, which appears to dovetail with paleoenvironmental proxies for the Dian basin. However, according to the *Shiji* records, the Han conquer of the Dian implied at least some level of political stability given that the former Dian King retained command of the lands (Watson 1961). Dian agriculture, as attested by archaeobotanical studies undertaken both at Hebosuo and other sites, such as Dayingzhuang and Xueshan, shows that the main crops at the basis of the Dian productive economy were wheat, rice, and millet, supplemented by the production of legumes such as soybean, possibly buckwheat and with the contribution of wild resources such as local fruits. As seen above, archaeobotanical remains from the 2016 Han samples show a continuity in composition and importance of plant species with both the late Dian period from the 2016 excavation, as well as the early Dian period reported from the 2014 excavation (Yang et al. 2021). This continuity may derive in part from the local soil and environmental conditions and/or constraints, given the peculiar vertical zonation of Yunnan, and this kind of mixed, dryland, and wetland regime represents the best choice for a rich agricultural production that takes advantage of water-rich lowlands and nearby highlands. The plains provide sufficient water resources for the cultivation of paddy rice, while the nearby hills provide a suitable environment for the cultivation of wheat and millets. This type of mixed cereal cultivation was introduced to the Dian area from at least the end of the second millennium BC (Yang et al. 2021). An additional reason for this continuation might be the effort made by the Han ruling class to integrate and find local solutions to maintain their political rule in new territories. By avoiding disrupting the local, pre-existing cultivation system, the new ruling class may have tried to prevent upheavals. Finally, the possibility of having an abundant food supply was also the driver for the Han migration into the fertile Dian Basin. Archaeological material culture retrieved from post-Dian sites also shows that although the Han Dynasty conquered the Dian in 109 BC, Dian local customs and lifeways continued for a period of time (Chiang 2012) until the first century AD coinciding with the abandonment of elite cemetery grounds.

Although there is not much change in overall composition of the agricultural assemblage from the Dian to Han periods, there is clear evidence indicating intensification of the cereal production in the Han period. Cereal crops increase in numbers during the Han (from 90 to 92.5% of the total recovered archaeobotanical remains), as well as in density (from 29.8 to 124.3 grains/L). This suggests an expansion and intensification of cereal cultivation that is possibly connected with migration-driven population growth. This is further supported by textual evidence found in the *Hou Han Shu* (Western Han Dynasty, Wang Mang period, AD 19), which records more 2000 hectares of new lands being cleared for cultivation during the protectorate of Wen Qi. The clearance of new land for agriculture is also attested by the increase in density of charcoal fragments from 2.7 to 7.2 g/L retrieved in the archaeological strata of the site and its surrounding. A recent study of lake sediments from the Dian Lake has detected an increased charcoal signature at the end of the first millennium BC (Xiao et al. 2020). The authors of the study have suggested this might represent an increased fire use, possibly linked to imperial land management policies.

Changes in relative proportion of cereal crops are also present from the Dian to the Han; although the ubiquity of rice and wheat both increase in the Han, the total numbers of remains show an increase of rice, in contrast with a marked decrease in wheat remains (Figs. 6 and 7). This might reflect a dislike of wheat by the Han migrant population, as attested by a slow uptake of wheat cultivation in the Central Plains until the end of the Han Dynasty (Boivin et al. 2012; Deng et al. 2020). Millet also shows a decrease in both ubiquity and frequency over time, in line with a declining trend that has been attested in other sites in Central Yunnan following the introduction of wheat in the mid-second millennium BC (Dal Martello 2022; Yang et al. 2021).

In addition to changes in cereal production, further changes can be detected in the increase in numbers and variety of fruit taxa in the Han period in comparison to the previous Dian period. Fruit taxa are present in very low quantities from sites in the Dian Basin preceding the occupation of Hebosuo, including Xueshan, Dayingzhuang, and Guangfentou (Dal Martello 2022; Wang et al. 2019; Dal Martello et al. 2021; Li and Liu 2016). This increase might indicate a preference to fruit by the Han population and possibly the beginning of fruit tree management practices, especially of peach trees. In the Lingnan area, homeland of the Nanyue Kingdom, there are recorded similar records of increase exploitation of Cucurbitaceae and Rosaceae fruits (Chen 2015).

### The question of irrigation and possible anthropogenic impact

The analysis of the weedy taxa composition from archaeobotanical samples also substantiates a shift in land use practices that is connected with wet rice cultivation introduced under the Han. Seeds of field weeds get incorporated into the archaeobotanical assemblage through the harvesting and crop processing activities carried out on site, as well as through food preparation. The presence of such weeds can thus help clarify the environment in which the crops they are associated with in the archaeobotanical assemblage were grown. Chronologically, dryland field weeds are more prevalent in the first phase of occupation of the site, while in the second phase, dryland weeds not only decrease and wetland weeds increase, but the overall variety of weed taxa also increases. This is usually linked with the clearance of new lands, but the particular increase in wetland weeds might mean a more intensive water management/irrigation practices were taking place, and/or the development of new agricultural production practices especially linked with new tools. Scholars believe that iron tools were employed in Yunnan since at least the Western Han period (202 BC–6 AD) with the production of pure iron tools and a marked increase in the number of agricultural tools recovered by the Eastern Han period (9–220 AD; see Lin 1963; Li and Zhou 2014). Such tools also impacted the precise plant harvesting practices, making it possible to harvest the whole plant from the roots. This practice would bring a higher number of weeds into the site, compared to harvesting the plant at the panicle, a method practiced with the claw sickle and attested during the Dian period. Previous archaeobotanical work at Dayingzhuang showed that earlier rice cultivation in Yunnan was mostly based on seasonal rain and alluvial activities from the nearby rivers (Dal Martello et al. 2021; Yang et al. 2021). However, from the Han period onward, rice cultivation may have shifted to a more intentional water management system, possibly with the wide-scale adoption of irrigation. The intensification of rice production, especially through paddy construction, terracing, and irrigation, can have a lasting impact on local vegetation and soil conditions. Analysis of palynological samples of lake sediment cores from the Dian Lake shows a notable increase in Poaceae pollen and charcoal density beginning in the first century AD (Xiao et al. 2020). The anthropogenic impact of a wet rice regime is also associated with an increase in soil erosion (e.g., uptick in magnetic susceptibility values) and the deposition of a red clay layer in lake sediments in both the northern and southern end of the lake (Hillman et al. 2019). These proxies suggest a more intensive land-use practice which corroborate temporally with the introduction of a more intensive cultivation regime documented in Han sources.

According to the *Houhan* Shu, Han officials described the Dian Basin as a vast agricultural land with abundant water resources, one that was well-suited for paddy rice cultivation. Historical accounts describe irrigation practices in the Dian Basin from at least the first century AD. The new prefecture established by the Han Emperor Wu in 109 BC, named the Yizhou Prefecture and largely consisting in the former Dian territory in Yunnan province, is described as having a large lake, identified with the modern-day Lake Dian, which was surrounded by salt wells, ponds, and vast agricultural fields. During the period of Wang Mang’s rebellion in AD 19, the central government appointed Wen Qi as the prefect of Yizhou, and he built the Bei pond in the Dian Lake area, to irrigate and develop agriculture (Fang and Li 1965). Intensive rice cultivation in paddy fields is much more labor intensive than growing wheat and millet. However, it also has the advantage of a much higher yield, which in turn can help prevent famine and related crisis. Han historical documents record that famines were a frequent occurrence in Yizhou Prefecture at the end of the first millennium BC. The most serious famine was recorded during the reign of Emperor Ling of the eastern Han Dynasty, during which time Jing Yi was the prefect of Yizhou. This crisis may have been amplified by population growth that was fueled by migration from the north. Short fall and rising demand drove up the price of rice until Jing Yi assumed office and stabilized the price of rice to previous levels (Chang 1985).

**Fig. 12:**
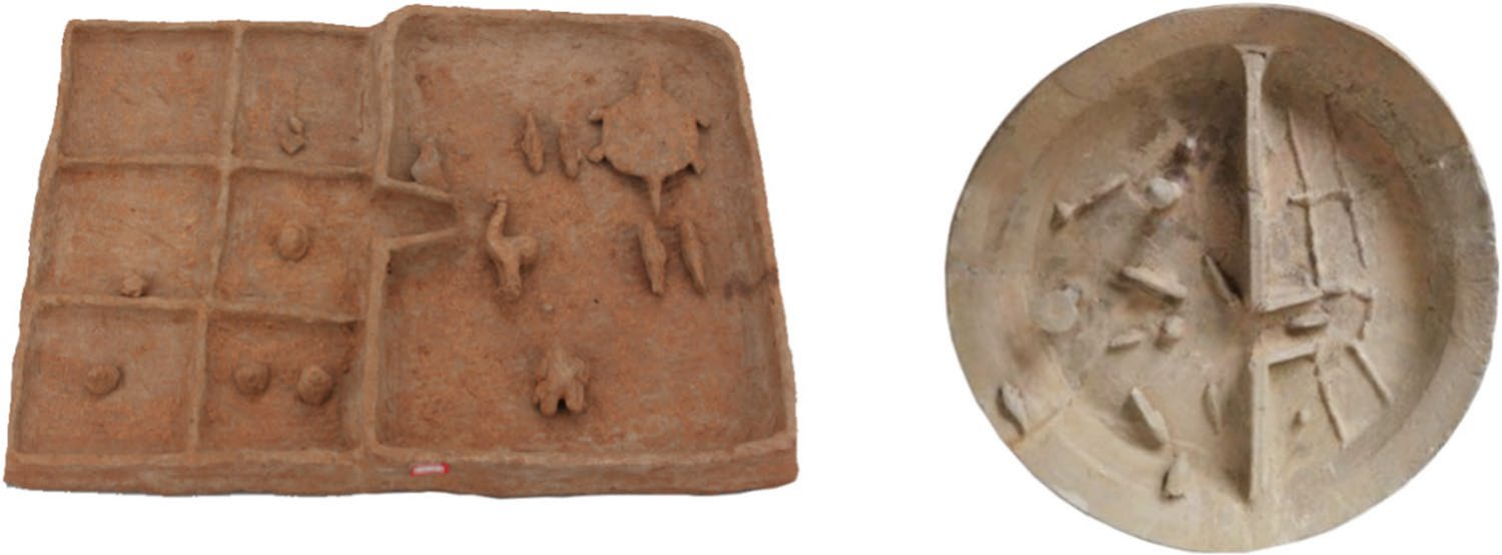
Models of rice paddy fields unearthed from Han period tombs in Yunnan. Left: Zhuowei Mt, Jinning, photo courtesy by Ms. Xuemei Yang from the Jinning district museum; right: Yangfutou cemetery in Kunming, photo by the Yunnan provincial museum (ynmuseum.org)

The construction of the Bei pond by Wei Qi is also attested by large clay models depicting paddy fields recovered from Han period tombs in the Dian Basin (Xiao 1994). These not only show paddy fields, but also details such as irrigation channels connecting them, the division and layout of the fields, the use of fish as nitrogen nutrient fixer, and the rearing of ducks, turtles, and fish on rice paddies (see Fig. 12). The frequency of iron farming tools in Han tombs also suggests that labor saving tools made for more effective and efficient tilling of clay soils. Similar clay models have also been recovered from the Erhai region, west from Dali, at the Dazhantun cemetery (Dali Zhou Wenwu Guanlisuo 1988; Zhang 1992) indicating that paddy rice cultivation of similar type than the one attested in the Dian basin was also present in Northwest Yunnan at about the same period. All of these visual and material sources illustrate an investment in a wide suite of technologies and infrastructures critical to supporting this farming system. These adoptions eventually transformed the local constitution of soils, water, and microbiota, changes which the paleoenvironment data do help confirm.

## Conclusion

Archaeobotanical analyses from the 2016 excavation season of Hebosuo provide the first direct evidence of agricultural production during the transitional period between the Dian and the arrival of the Han in the Dian Basin, in Central Yunnan. Although the same staple crops (rice and wheat) continued to be cultivated during this transition period, the weed assemblage shows that new agricultural regimes associated with wet or irrigated systems were being introduced. These practices may be tied to both migration and population pressures and attested state efforts to intensify agricultural production in the region. Other important economic species include soybean and adzuki bean. Chenopodium was retrieved in high quantities and this might indicate it was actively exploited; finally, Sichuan pepper grains were also retrieved. However, the early exploitation and domestication trajectory of these species are not well understood and future studies should address this issue. At present, archaeobotanical evidence indicates cultivation of similar staple crops with the arrival of the Han; some intensification of the agricultural production can be attested, especially in regard to the irrigation of rice, and diversification of local wild taxa, which corroborate with the increase in charcoal volume and grass pollen, as well as soil erosion rates, detected in lake sediments by previous studies (Hillman et al. 2019; Xiao et al. 2020). Future targeted excavation of post-Dian sites in central Yunnan will also help clarify the degree of intensification of the productive economy that is presently attested at Hebosuo.

## Supplementary Information

Below is the link to the electronic supplementary material.

Supplementary file1 (XLSX 40 KB)

Supplementary file2 (PDF 341 KB)

## Data Availability

All data generated and analyzed during this study are included in this published article (and its supplementary information files).
