# Supplementary material for: Food production and agricultural systems on the southwestern frontier of the Han Empire: archaeobotanical remains from the 2016 excavation of Hebosuo, Yunnan
Source: Archaeol Anthropol Sci. 2023 May 4;15(5):71. doi: 10.1007/s12520-023-01766-9 (PMC10160161; doi:10.1007/s12520-023-01766-9)
Supplement: Supplementary file 2 — Supplementary file2 (PDF 341 KB) [file 12520_2023_1766_MOESM2_ESM.pdf]

Supplementary material S2.

Individual grain measurements on main species from the 2016 Hebosuo samples.

Wheat- *Triticum aestivum*

Grain n.	Time Period	Type	Lenght in mm	Width in mm	L:W
**1**	Dian	2	3.347	2.636	1.269727
**2**	Dian	2	3.461	3.066	1.128832
**3**	Dian	2	3.264	2.456	1.32899
**4**	Dian	2	3.483	2.756	1.263788
**5**	Dian	2	2.861	2.177	1.314194
**6**	Han	2	3.741	3.347	1.117717
**7**	Han	2	3.366	2.833	1.18814
**8**	Han	2	3.197	2.347	1.362164
**9**	Han	2	3.22	2.416	1.332781
**10**	Han	2	2.489	1.957	1.271845
**11**	Han	2	3.453	2.616	1.319954
**12**	Han	2	3.644	2.915	1.250086
**13**	Han	2	2.832	2.239	1.26485
**15**	Han	2	3.676	2.832	1.298023
**16**	Han	2	3.57	2.618	1.363636
**17**	Han	2	2.923	2.455	1.190631
**18**	Han	2	4.159	3.023	1.375786
**35**	Han	2	3.353	2.593	1.293097
**20**	Han	2	3.345	2.539	1.317448
**21**	Han	2	3.169	2.678	1.183346
**22**	Han	2	3.12	2.711	1.150867
**23**	Han	2	3.284	2.827	1.161655
**24**	Han	2	4.027	3.83	1.051436
**25**	Han	2	4.164	3.005	1.385691
**26**	Han	1	4.005	2.425	1.651546
**27**	Han	1	3.949	2.342	1.686166
**28**	Han	1	4.126	2.567	1.607324
**29**	Han	1	3.513	2.159	1.627142
**30**	Han	1	4.03	2.484	1.622383
**31**	Han	1	4.08	2.207	1.848663
**32**	Han	1	3.741	2.318	1.613891
**33**	Han	1	3.822	2.377	1.607909
**34**	Han	1	3.376	2.201	1.533848
**36**	Han	1	3.46	1.97	1.756345
**37**	Han	1	3.535	2.416	1.463162
**38**	Han	1	2.779	1.565	1.775719
**39**	Han	1	3.872	2.451	1.579763
**14**	Han	1	3.299	1.938	1.70227
**19**	Han	1	3.224	2.213	1.456846

Rice- *Oryza sativa*

Grain n.	Time period	Context	Length in mm	Width in mm	Thickness in mm	L:W
**1**	Dian	T2⑰b-2-3	3.853	2.148	1.728	1.793761639
**2**	Dian	T2⑰b-2-2	3.627	2.437	1.921	1.488305293
**3**	Dian	T2⑰b-2-1	4.368	2.454	2.079	1.7799511
**4**	Dian	T1⑰c-1	4.102	2.42	2.094	1.695041322
**5**	Dian	T1⑰b-1-2	4.24	2.323	1.91	1.825226001
**6**	Dian	T1⑰b-1-1	4.436	1.929	1.684	2.299637118
**7**	Dian	T1⑰b-1	4.053	1.334	1.087	3.038230885
**8**	Dian	T1⑰a-1-3	3.992	2.131	1.678	1.873298921
**9**	Dian	T1⑰a-1-2	4.232	2.327	1.707	1.818650623
**10**	Dian	T1⑰a-1-1	5.214	3.398	2.51	1.534432019
**11**	Han	T2⑭-1	4.513	2.338	2.048	1.930282293
**12**	Han	T1T2⑨： 8-1-3	3.912	2.799	1.995	1.397642015
**13**	Han	T1T2⑨： 8-1-2	4.829	2.699	2.11	1.789181178
**14**	Han	T1T2⑨： 8-1-1	4.615	2.458	1.871	1.877542718
**15**	Han	T1⑯-8	5.665	2.671	2.266	2.120928491
**16**	Han	T1⑯-3	4.47	1.942	2.018	2.301750772
**17**	Han	T1⑭-3	4.181	2.108	1.447	1.983396584
**18**	Han	T1⑭-2	4.324	1.778	1.341	2.431946007
**19**	Han	T1⑬-4-3	4.188	2.756	2.209	1.519593614
**20**	Han	T1⑬-4-2	6.264	3.109	2.268	2.014795754
**21**	Han	T1⑬-4-1	4.478	2.21	2.067	2.026244344
**22**	Han	T1⑪-2	5.127	2.968	2.084	1.727425876
**23**	Han	H2-2-3	4.781	2.553	2.053	1.872698786
**24**	Han	H2-2-2	4.158	2.48	1.788	1.676612903
**25**	Han	H2-2-1	5.401	2.106	2.187	2.564577398

Foxtail millet- *Setaria italica*

Grain no.	Time Period	Context	Length in mm	Width in mm	Thickness in mm	L:W
**1**	Dian	T1⑰c-1-1	1.027	1.157	0.848	0.88764
**2**	Dian	T1⑰c-1-2	0.954	1.022	0.734	0.933464
**3**	Dian	T1⑰c-1-3	1.056	0.898	0.664	1.175947
**4**	Dian	T1⑰c-1-4	0.994	0.825	0.833	1.204848
**5**	Han	T1⑮-3	1.423	0.997	0.709	1.427282
**6**	Han	T1⑬-4	1.478	1.516	1.254	0.974934
**7**	Han	H2-1-1	1.356	1.143	0.936	1.186352
**8**	Han	T2⑭-1	1.032	0.861	0.668	1.198606

Peach- *Prunus persica (Amygdalus persica*)

Grain no.	Length in mm	Width in mm	Thickness in mm
**1**	2.27	1.84	1.79
**2**	2.01	1.64	1.34
**3**	2.50	1.83	1.40
**4**	2.38	1.67	1.30
**5**	2.89	2.00	1.61
**6**	2.43	1.88	1.48
**7**	2.00	1.53	1.28
**8**	1.89	1.52	1.33
**9**	2.51	1.79	1.17
**10**	1.84	1.64	1.36
**11**	2.46	1.75	1.40
**12**	1.99	1.58	1.41
**13**	2.26	1.60	1.38
**14**	2.16	1.65	1.32
**15**	1.95	1.56	1.18
**16**	2.28	1.70	1.43
**17**	1.95	1.49	1.23
**18**	2.04	1.47	1.22
**19**	2.02	1.58	1.26
**20**	2.51	1.89	1.47
**21**	2.09	1.50	1.07
**22**	2.18	1.78	1.53
**23**	2.37	1.80	1.45
**24**	1.80	1.54	1.27
**25**	1.89	1.55	1.30
**26**	1.92	1.58	1.18
**27**	1.83	1.37	1.13
**28**	2.01	1.61	1.26
**29**	2.43	1.61	1.31
**30**	1.85	1.60	1.30
**31**	1.90	1.31	1.09
**32**	1.84	1.51	1.24
**33**	2.11	1.70	1.27
**34**	1.97	1.48	1.20
**35**	2.16	1.58	1.17
**36**	2.00	1.55	1.27
**37**	2.08	1.52	1.14
**38**	2.34	1.72	1.36
**39**	2.47	1.79	1.37
**40**	2.13	1.66	1.43
**41**	2.12	1.78	1.25
**42**	2.40	1.82	1.38
**43**	1.96	1.59	1.31

**44**	2.06	1.69	1.24
**45**	2.13	1.61	1.35
**46**	1.80	1.51	1.13
**47**	2.13	1.66	1.56
**48**	2.16	1.72	1.30
**49**	2.34	1.74	1.42
**50**	2.08	1.55	1.25
